# Working with the grain: uncovering sorghum quality regulatory networks

**DOI:** 10.1093/jxb/erag108

**Published:** 2026-04-15

**Authors:** Carla F López-Gómez, Karen Massel, Ian D Godwin

**Affiliations:** Queensland Alliance for Agriculture and Food Innovation, The University of Queensland, Brisbane, QLD, Australia; Queensland Alliance for Agriculture and Food Innovation, The University of Queensland, Brisbane, QLD, Australia; Queensland Alliance for Agriculture and Food Innovation, The University of Queensland, Brisbane, QLD, Australia

**Keywords:** Protein digestibility, protoplast transient overexpression, regulatory network, sorghum, starch and protein accumulation, transcriptomics

## Abstract

This article comments on:

**Séne M, Calatayud C, Berger A, Soriano A, Richaud F, De Bellis F, Sotillo A, Rios M, Bonicel J, Mameri H, Pot D, Terrier N.** 2026. Integrative transcriptomic and functional analyses reveal candidate transcription factors associated with sorghum grain quality. Journal of Experimental Botany **77**, 2349–2366. https://doi.org/10.1093/jxb/erag015

This article comments on:

**Séne M, Calatayud C, Berger A, Soriano A, Richaud F, De Bellis F, Sotillo A, Rios M, Bonicel J, Mameri H, Pot D, Terrier N.** 2026. Integrative transcriptomic and functional analyses reveal candidate transcription factors associated with sorghum grain quality. Journal of Experimental Botany **77**, 2349–2366. https://doi.org/10.1093/jxb/erag015

**Co-expression profiles and protoplast transient overexpression assays are beginning to unravel the complex regulatory network governing sorghum grain quality, revealing key transcription factors (TFs) that control starch and protein biosynthesis, and digestibility (Séne *et al*., 2026). This integrated approach provides new targets to accelerate improvement of the nutritional value of sorghum through plant breeding and genetic engineering.**

Improving grain quality is one of the primary goals of crop breeding, particularly in sorghum, where its potential is devalued by low protein content and digestibility. While established methods such as quantitative trait locus (QTL) mapping or single nucleotide polymorphism (SNP) arrays identify associated genomic regions, these techniques only provide positional information based on correlation, offering little insight into the underlying molecular mechanisms. As grain quality is a complex trait highly influenced by development and environmental factors, it necessitates direct and quantitative techniques. Addressing this gap, Séne *et al*. (2026) delineate a strategy to uncover a component of this intricate regulatory network by implementing transcriptomic analysis and cutting-edge protoplast transient overexpression assays.

The objective is clear—what is the best way to understand how genetic networks are regulated to enable future genetic improvements? The power of transcriptomics relies on comparing gene co-expression across treatments and can be enriched by its interaction with distinct environments and genetic backgrounds. Exploring these data infers regulatory relationships and highlights hub genes (encoding potential TFs) that control entire functional modules (Wolf, 2013; Evans, 2015). Séne *et al*. (2026) reveal two major modules determining grain development, namely protein and starch accumulation, and limitations to protein digestibility.

Predictably, protein and starch accumulation modules are enriched with genes encoding different kafirins, which account for 80% of the protein content in sorghum grain, and starch-related enzymes, respectively. Additionally, these modules feature other genes involved in kernel development, lipid biosynthesis (starch granules include small amounts of lipids), and protein trafficking. Moreover, an intimate intricacy in the regulation of protein and starch biosynthesis is revealed. This is not surprising, given that both processes share common metabolic precursors and are physically interconnected within the endosperm (Bean *et al*., 2019). From the perspective of a breeder or a genetic engineer, however, the identification of unlinked genes would be of great value. In sorghum, high starch content is preferred for the bioenergy sector, whereas high protein content retains considerable value for the animal feed market and, potentially, for the human food sector.

It should be noted that higher protein content would only be beneficial if it is accompanied by improved digestibility. Within the digestibility loss module, an interesting thioredoxin family involved in disulfide bond formation appears (Séne *et al*., 2026). This finding is notable as many storage proteins are cysteine rich, requiring assistance to be properly folded. Indeed, the thioredoxin family has been reported to regulate seed storage protein accumulation and composition in maize and rice (Li and Larkins, 1996; Takemoto *et al*., 2002), and it represents a promising target for future efforts to modulate protein digestibility in sorghum.

Despite the power of transcriptomics, its interpretation requires careful consideration as results are often constrained by data quality, coverage, and the fact that mRNA abundance does not always correlate with protein levels, particularly for TFs (Vogel *et al*., 2010; Leong *et al*., 2025). Addressing this challenge, Séne *et al*. (2026) performed a follow-up experiment using a protoplast-based transient expression system (PTES). This system allows the delivery and expression of selected genes within protoplasts over a short duration. Compared with stable transformation, PTES offers a rapid and economical platform to screen numerous transgene events. Using this technique, pre-selected TFs are ectopically expressed, enabling the authors to infer the first grain development network for sorghum available in the literature (Séne *et al*., 2026).

The strategic combination of transcriptomics with protoplast transient overexpression holds significant potential to increase our understanding of other partially understood, or completely unknown, mechanisms in plants such as stress adaptation, specialized metabolism, or biotic resistance. While transcriptomic analysis generates likely gene co-expression networks, PTES validates the regulatory activity of candidate genes. Ultimately, an experimental validation using proteomics or gene silencing would help to establish the most relevant connections (Fig. 1).

**Fig. 1.**
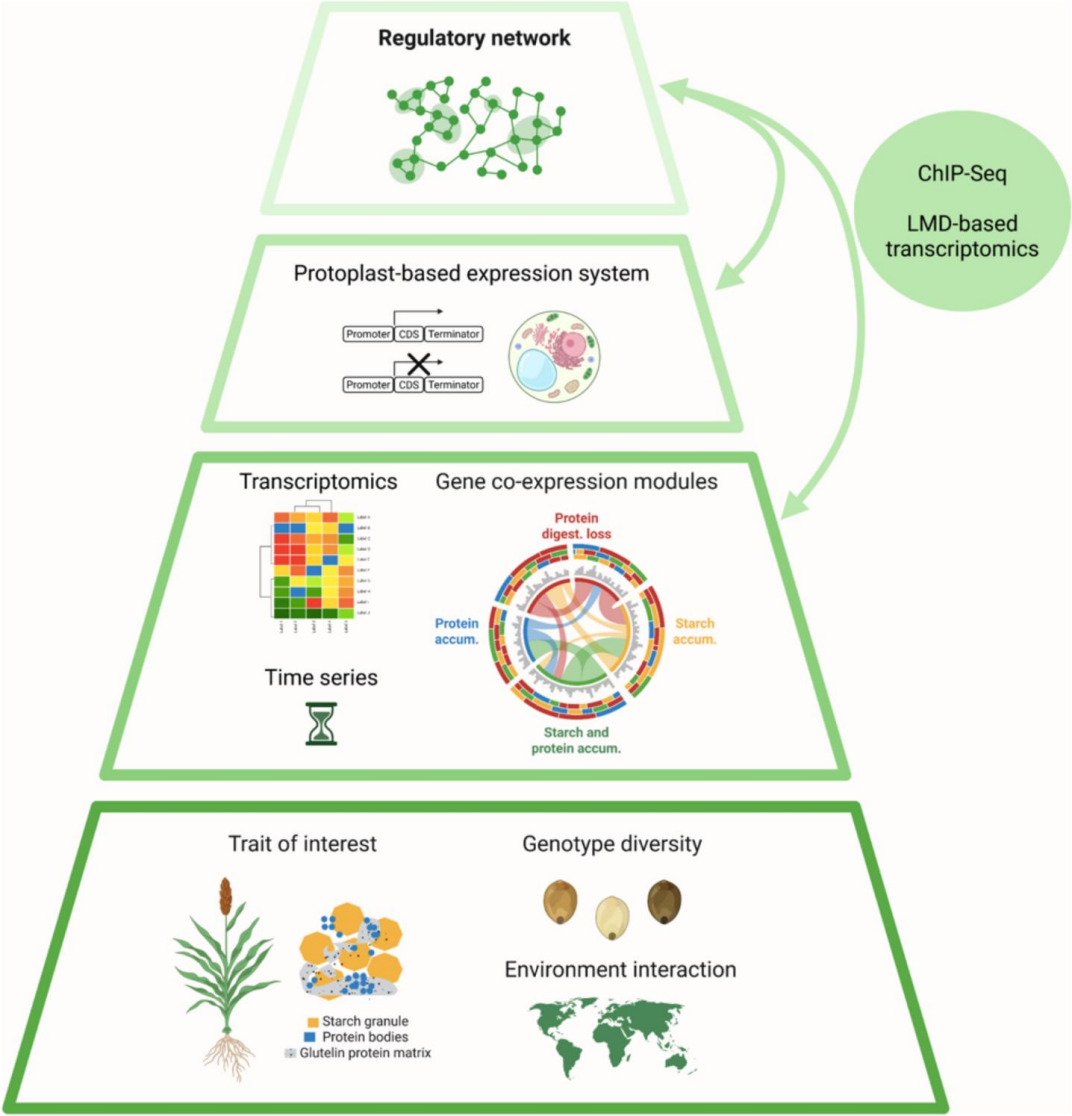
Pyramidal workflow to unravel regulatory networks in crops. Séne *et al*. (2026) delineate an innovative workflow that integrates transcriptomic data with a protoplast-based transient expression system (PTES) to decipher the first regulatory network governing grain development in sorghum. From bottom to top: gathering transcriptomic data of grain quality (trait of interest) across distinct environments and genetic backgrounds; identification of regulatory relationships and functional modules (*i.e.,* protein digestibility loss, protein accumulation, starch accumulation, and starch and protein accumulation); validation of candidate hub genes using PTES, ChIP-Seq, and laser microdissection-based transcriptomics; and construction of a refined regulatory network governing grain quality in sorghum.

PTES has also proven invaluable for identifying subcellular localization, elucidating protein–protein interactions, and uncovering cellular functions across several species including Arabidopsis, wheat, grapevine, rice, melon, and potato (Chen *et al*., 2023). Furthermore, the utility of this approach extends to other potential applications in plant biotechnology. For example, to test the efficiency of different Cas9 variants, gRNAs or candidate genes prior to committing to stable transformation, which is time and resource consuming. PTES could be the alternative approach in cereals to leaf infiltration in tobacco.

Nevertheless, a major issue regarding PTES requires discussion. Mesophyll cells become dedifferentiated upon cell wall removal, and thus can be employed to express genes that are normally active in other cell types and tissues (Yoo *et al*., 2007). Even though tissue dependency is alleviated, PTES still exhibits other limitations as it may ignore crucial factors such as epigenetics, chromatin remodelling, and presence/absence variations (*i.e.,* genomic sequences found in some individuals but missing in others within the same species), which is notably prevalent in sorghum (Zhang *et al*., 2014). Thus, the integration of other advanced approaches such as ChIP-seq with spatially resolved transcriptomics, such as laser microdissection-based transcriptomics, could be used to identify master switches. Moreover, confirming whether the newly identified interacting TFs are also differentially expressed would establish a valuable feedback loop to refine a more comprehensive network (Fig. 1).

Overall, this represents a highly promising strategy to rapidly elucidate several complex molecular mechanisms. This approach opens the door to identify an unlimited number of novel targets, which would significantly complement current global efforts for crop improvement.
